# Evaluating ChatGPT’s Effectiveness for Arabic Dry Mouth Patient Education

**DOI:** 10.3390/healthcare14172681

**Published:** 2026-08-24

**Authors:** Abdullah Mohamed Alsoghier

**Affiliations:** Department of Oral Medicine and Diagnostic Sciences, College of Dentistry, King Saud University, Riyadh 11545, Saudi Arabia; aalsoghier@ksu.edu.sa; Tel.: +966-11-4677423

**Keywords:** oral medicine, salivary gland diseases, health literacy, patient education, generative artificial intelligence

## Abstract

**Background/Objectives**: The present study aimed to assess the understandability and actionability of Arabic text generated by a large language model for commonly searched Arabic queries on dry mouth. **Methods**: Using Google Trends, the top 10 searches worldwide related to ‘oral dryness’ were entered in OpenAI’s Generative Pretrained Transformer 5.1. Generated texts were achieved independently. Assessments were performed using the Patient Education Materials Assessment Tool (PEMAT) to evaluate the content, word choice, and style. **Results**: Causes, symptoms, and treatment of dry mouth were the most common dry-mouth-related queries. The highly temporal distribution of search interests among Arabic-speaking countries peaked between 2020 and 2021, then remained high throughout 2023, before declining in November 2025. The mean PEMAT understandability and actionability scores were 89% and 80%, respectively. It was notable that all generated responses lacked visual aids, which could have made the content difficult to understand and insufficient for acting on the information. Moreover, the formal Arabic form of ‘causes of dry mouth’ with a glottal stop yielded lower actionability scores (60%) than the informal Arabic form (80%). **Conclusions**: Clinicians could actively supplement clinic-based discussions with advice on using large language models to help patients recognise dry mouth symptoms and improve self-care. Also, they could improve their effective adoption by clearly explaining expectations, limitations, and language/cultural differences when adopting these models.

## 1. Introduction

It is not uncommon these days to discuss a medical practice and health education topic without explicitly highlighting the relevance and applications of large language models (LLMs) in context. These computer-based programmes aim to generate text that appears human-generated in response to a search query or ‘prompt’, and have a significant impact on and offer opportunities in modern medical practices and health education [1,2]. These models showed variability in information accessibility, with some demonstrating concerns about misinterpretation of health records, limited comprehension of International Classification of Diseases codes, and inaccurate predictions of clinical outcomes [3,4]. Notably, several LLMs have been discussed for clinical tasks, such as ChatGPT-4, Baichuan, Claude-3.5, InternLM, Llama, Med42, MEDITRON, Mistral, and Qwen [4].

The role of LLMs has been widely discussed in patient education, providing personalised health advice and answering patient and community queries about their own health [1]. This is highly essential, especially for individuals with limited health literacy who have difficulty reading and comprehending the information needed for their own healthcare decision-making [5]. Studies showed that OpenAI’s Chat Generative Pretrained Transformer (ChatGPT) has indeed improved patients’ comprehension and self-confidence, especially among minority groups and older adults [5]. There is also an increasing trend in utilising it to generate patient educational materials in oncological and ophthalmological settings [6]. It also outperformed other available models in terms of information reliability and understandability for educational information on cardiovascular and neurological conditions, respectively [7]. Compared to other LLMs, ChatGPT models were highly adopted in the literature to address common patient questions, improve existing materials, translate materials from other languages, simplify medical information, provide lifestyle advice, and promote health literacy [8].

The rationale for using LLMs is further emphasised by increased post-appointment visits to the health portal and by frequent medical record reviews to understand what was discussed and determined during a clinical visit [5]. Adopting LLMs for patient education can help maintain tailored, high-quality healthcare services and effective patient–clinician communication [9]. Its high accessibility and ease of use can complement the sometimes time-limited clinical discussions [10]. Moreover, it could help to meet patients’ information needs, as many seek additional health information after clinical visits to help them interpret what was discussed with their clinician and recall the next management steps [5,11,12].

The subjective sensation of dry mouth (xerostomia) is a common health complaint with considerable socioeconomic burden on healthcare systems worldwide [13,14]. It is often associated with objective clinical findings of salivary hypofunction, and may lead to several functional burdens (e.g., chewing, tasting, swallowing) and significantly increase the risk of oral and dental diseases [13]. Causes of xerostomia include medications, cancer therapy, autoimmune diseases, diabetes mellitus, and increased ageing [15]. Notably, epidemiological studies showed varied prevalence rates across countries and continents, ranging from 13 to 22% [15,16], with rates in Middle Eastern countries reaching up to 42% [17].

Aside from addressing the patient’s information and care needs, clinicians may help patients access tangible self-care resources and identify reliable sources of further health information to improve disease outcomes, promote early detection, and adopt positive health behaviours [12]. Recent studies have raised concerns about the quality of web-based oral health information available to patients and the public—those related to the Arabic language were no exception [18]. The Arabic language is one of the most spoken languages worldwide, with around 500 million speakers mainly in Middle Eastern and North African countries [19,20]. It is also a language that uses different morphological forms derived from a single word, with diverse uses of affixes and prefixes, unlike English [20].

The Patient Education Materials Assessment Tool (PEMAT) is a widely used instrument to assess the quality of written and audiovisual health information [21,22]. It specifically addresses whether readers can comprehend what they read (understandability) and act upon it (actionability) [21]. Little is known about the effectiveness of LLMs as health education tools for Arabic-speaking populations, particularly for oral health complaints. These tend to be chronic and often impact one’s health-related quality of life. Therefore, the present work aimed to assess whether a layperson could understand LLM-generated text on common queries about dry mouth and act on the information provided using PEMAT.

## 2. Materials and Methods

### 2.1. Study Methods

The present study qualitatively assessed ChatGPT-generated text for commonly searched Arabic terms related to dry mouth. Google Trends (https://trends.google.com/trends/) was accessed on 23 November 2025 using a private Safari browser [website version of GPT 5.1 (OpenAI, San Francisco, CA, USA), Safari browser version 18.6 (macOS Ventura, Apple Inc., Cupertino, CA, USA), in incognito mode, without signing in for each searched term]. Following a previously adopted query extraction method [23], the Arabic analogue of ‘dry mouth’ was searched worldwide, with a time limit from 2004 to the present, across all categories, and ‘web search’ was selected. Worldwide searches, rather than country-based ones, were performed to reflect the language’s global use beyond Middle Eastern countries. This also ensured a comprehensive analysis of Arabic analogies for ‘dry mouth’ across dialects (e.g., Gulf or North African Arabic) [24].

Related queries were those relevant to dry mouth, with interest by city and subregion where the searched term/s were used during the specified time frame. Based on Google Trends analysis, a popularity score (0–100%) proportional to the percentage of residents in that area who searched for the term was generated. The lower the score, the fewer the people interpreted as searching for such a term [23]. Following this, each dry-mouth-related term was entered into ChatGPT using the same private browser on a separate day (24 November 2025), and the generated information was archived as printed files for understandability and actionability assessments (Supplementary Files S1 and S2).

### 2.2. Understandability and Actionability Assessments

The PEMAT for printed materials [21] was used to score both understandability (*n* = 16) and actionability (*n* = 5) based on whether the criteria were met: agree (1 point), disagree (0 points), or not applicable (the question was excluded from the total possible points). The used version of PEMAT previously showed strong reliability (internal consistency) and validity (content, face, and construct) in assessing the quality of written information [21]. The total obtained scores were then divided by the total scores and multiplied by 100 for a percentage. An acceptable cut-off level was defined as greater than 70% for both criteria [15] (Table 1).

To ensure the reliability of PEMAT assessments, one assessor—an expert in oral medicine with a PhD, with expertise in allied health instrument psychometrics and patient education—initially completed all PEMAT assessments of the generated materials. Then, another rater with a similar background completed the same task independently. Inter-rater reliability was then calculated using Cohen’s Kappa and agreement percentages [22]. Inter-rater reliability indicated substantial agreement (κ = 0.767, *p* < 0.001) and high agreement (94.1%). There was disagreement regarding only one PEMAT item (whether the printable material provided a summary); thus, a consensus was made between the assessors to follow the scoring guidelines that grade the short materials as not applicable [21].

### 2.3. Data Analysis and Quality Assurance

Descriptive analysis (mean, standard deviation, and percentages) was performed for the study variables on each assessed website. Data were initially entered and coded in Microsoft Excel (v. 16) and analysed in IBM SPSS Statistics (v. 29). Values are presented as frequencies (percentages). Percentages are calculated based on the total number of evaluated queries (*n* = 10).

## 3. Results

The most searched queries related to dry mouth were causes of dry mouth; symptoms of dry mouth; treatment of dry mouth; dry throat; dry mouth and throat; and dry mouth during sleep. Less common related queries also included causes of dry throat and dry nose (Table 2).

**Figure 1:**
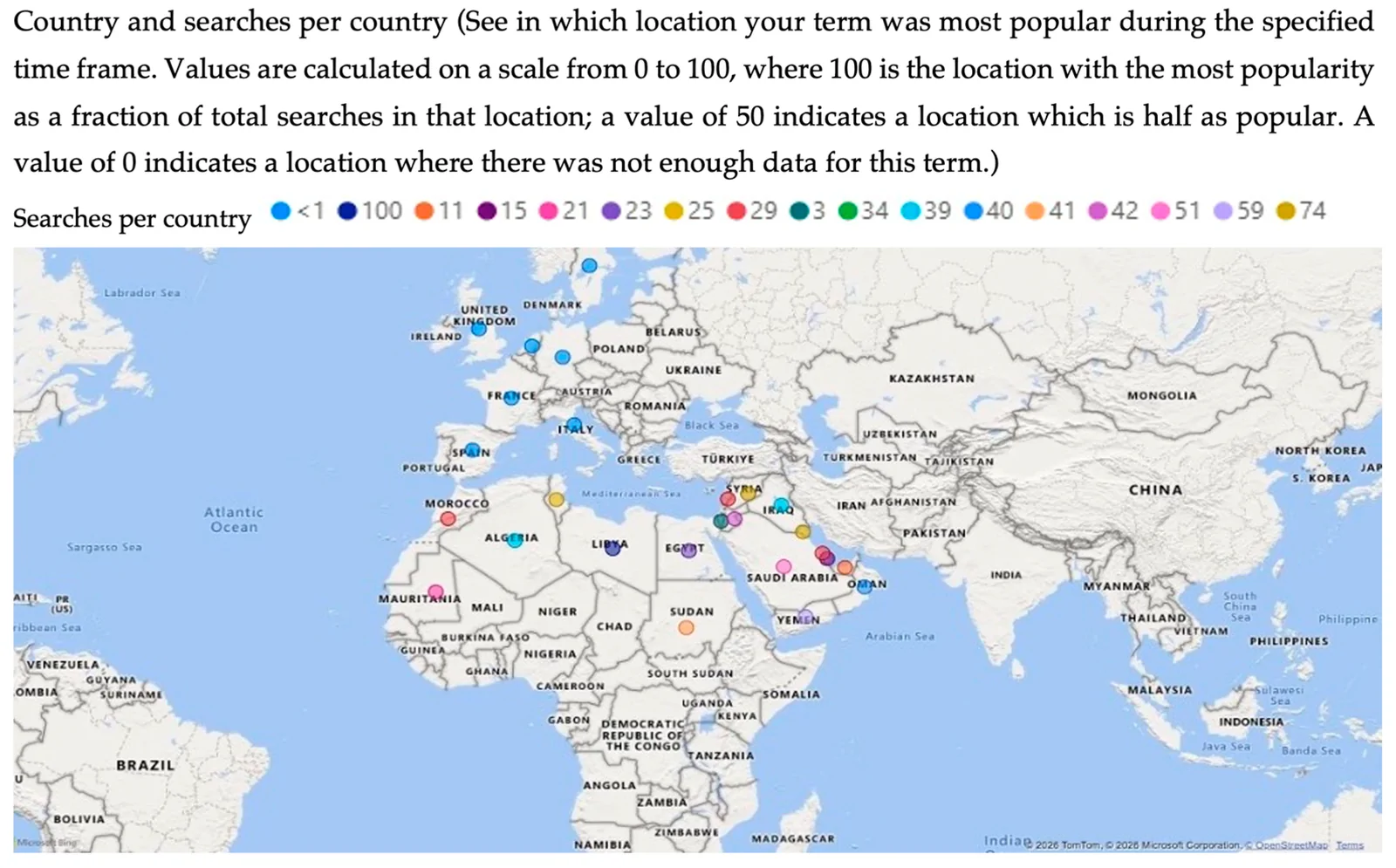
Country-wise distribution of Google searches related to dry mouth. Note: These points demonstrate improvements by Google’s geographical assignment from 1 January 2011, as well as the data collection system from 1 January 2016 and 1 January 2022, respectively.

The global distribution of dry-mouth-related search interest, as captured by Google Trends, is shown in Figure 1.

**Figure 2:**
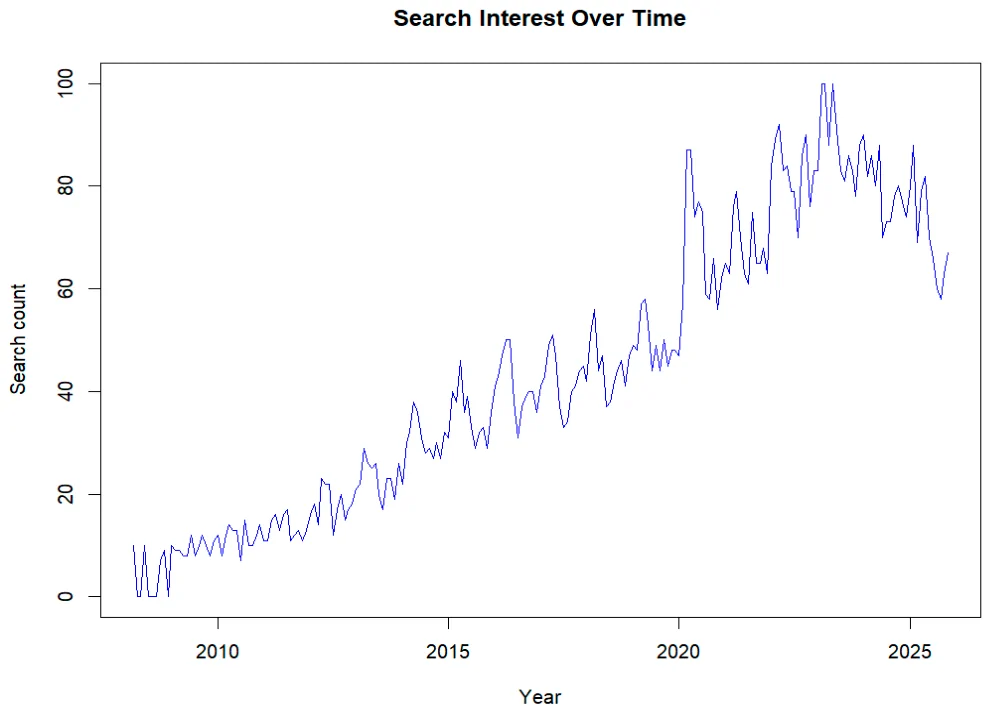
The search count for dry-mouth-related information from 2010 to 2025.

Based on a 0–100 popularity score on Google Trends, Libya, Syria, Yemen, and Saudi Arabia scored 50% or higher. On the other hand, Western Sahara, Jordan, Algeria, Sudan, Oman, and Iraq were at 50% or below (Supplementary File S3). Within Saudi Arabia’s regions, the highest interest trend was in Aseer Province, followed by Jazan, Aljouf, Hail, Alqassim, Almadinah, Najran, Albahah, Tabuk, and the Northern Border. Figure 2 shows the search count for dry-mouth-related information from 2010 to 2025, indicating a gradual increase, peaking between 2020 and 2021. Thereafter, it continued to increase through 2023, but a steady decline was demonstrated until 2025. The analyses also demonstrated a surge in searches that occurred in March and April 2020, which recurred in 2021, 2022, 2023, 2024, and 2025 (Supplementary File S4).

**Figure 3:**
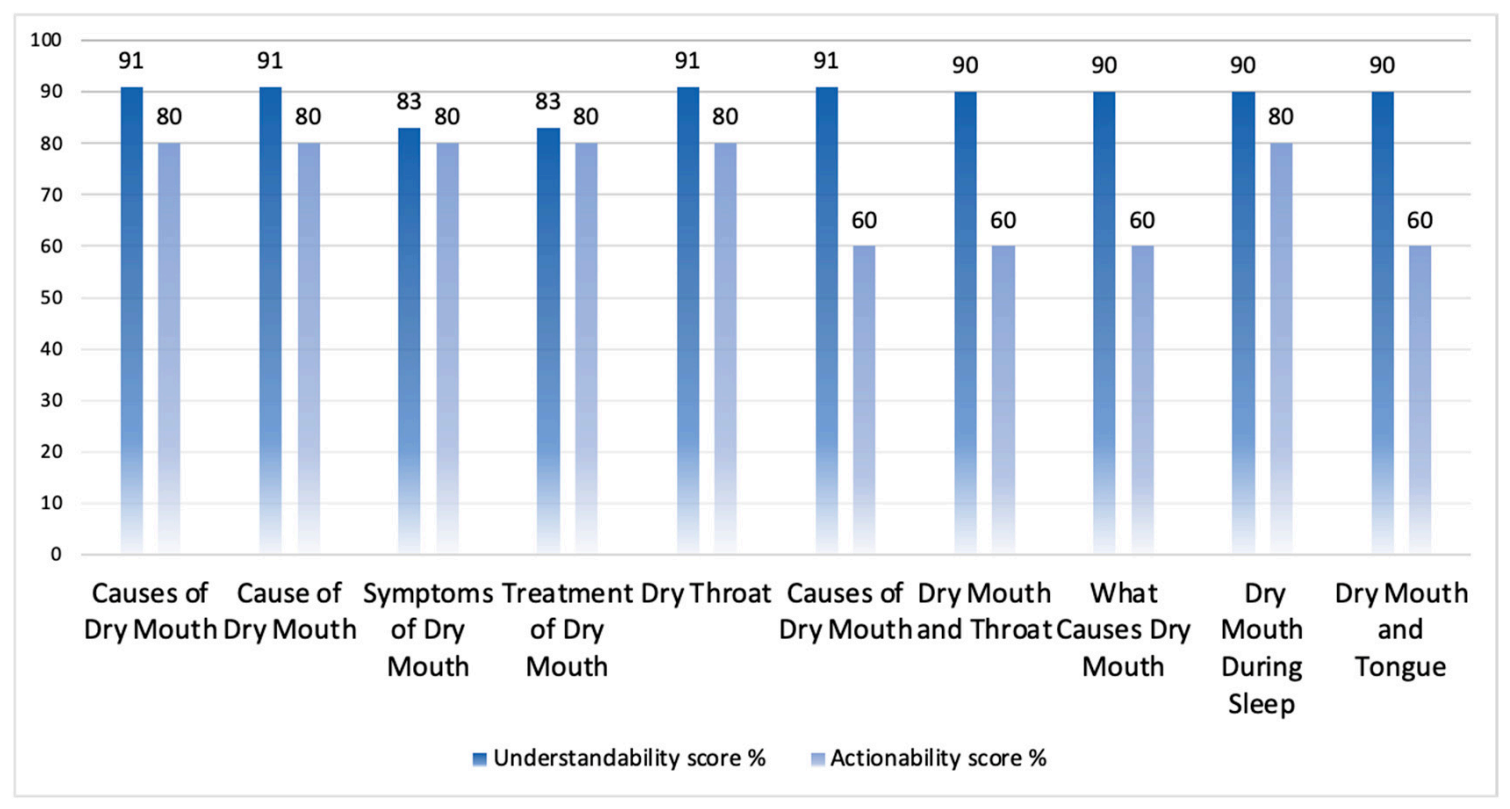
The PEMAT scores for ChatGPT-generated Arabic dry mouth content.

Regarding the PEMAT analysis, the total understandability and actionability scores were 89.7% (±3.2) and 80% (±10.3), respectively. Notably, content related to 8 of the 10 searched terms scored more than 90% for understandability. Exceptions were observed regarding the symptoms of dry mouth and in its treatment, both of which were scored at 83%. In contrast, content related to four of these terms scored below the actionability cut-off (<70%) (Figure 3 and Table 3).

Regarding understandability items with low scores, all searched terms lacked visual aids, which would have made the content easier to understand (PEMAT-15), and two of these lacked sections with informative headers (PEMAT-2). Similarly, none of the assessed content contained visual aids, illustrations, or photographs (PEMAT-16, 17, 18, and 19). Also, only 3 of the 10 terms provided a summary (PEMAT-11), and the remaining terms were very short (less than 2–3 paragraphs and no longer than one page). In turn, all results had an evident purpose (PEMAT-1), presented no distracting information or content (PEMAT-2), used everyday language (PEMAT-3), used medical terms only to familiarise the reader (PEMAT-4), and used active voice (PEMAT-5). Moreover, all content met the understandability criteria related to breaking information into concise sections (PEMAT-8), presenting information in a logical sequence (PEMAT-10), and using visual cues to draw attention to key points (PEMAT-12). Similarly, none of them expected the user to perform calculations (PEMAT-7).

For actionability, the lack of visual aids is reflected in the obtained scores for all terms, demonstrating that none included visual aids that made it easier to act on the given instructions. Furthermore, it was notable that content related to causes of dry mouth, dry mouth and throat, what causes dry mouth, and dry mouth and tongue did not present a tangible tool (e.g., menu planners, checklists) to help the reader take action. Nevertheless, all showed at least one action the user could take, addressed the reader directly when describing actions, and broke down any action into manageable steps (PEMAT-20, 21, and 22).

Analysis also showed that searching ‘causes of dry mouth’ in the Arabic language without a glottal stop (hamza) was the most searched term, whereas the more formal use of this stop was the sixth (Supplementary Files S1 and S2). Also, both produced different outputs: the former included no formal summary or definition, addressed the reader directly, and provided simple steps for self-management, whereas the latter did not. This led to a favourable PEMAT actionability score, indicating greater reader actionability (80% compared to 60%, respectively). It also presented a note to discuss age and symptoms, highlighted medications as a potential reason, and suggested appropriate treatment. Moreover, it showed fewer reasons (8 versus 10 reasons), with a lack of notes on salivary gland disorders and alcohol/caffeine drinking.

## 4. Discussion

The present work provided a detailed analysis of the potential role of an AI model in answering common patient search queries related to widely reported worldwide oral health complaints. It also adopted a recognised method to identify what patients search for regarding dry mouth and to assess whether the generated text is comprehensible and actionable. It also shows language-related variations in oral health information that have not been clearly identified, indicating that different translations of the same English search term may yield different results and actionability scores. Moreover, Google Trends was used to highlight the number of searches for dry mouth over time or by geography [25]. It is widely considered as a geographical and temporal indicator for health information-seeking behaviours on the internet [24,25,26]. It may also indicate the reach of a health campaign and awareness of a health concern (e.g., Cancer Day) [26]. Studies on digital health literacy in Middle Eastern and North African Arab countries showed varied reasons for searching the web, including seeking health information, participating in groups, and checking symptoms [27,28]. The latter was likely encountered in the present study, as diabetes and dry mouth symptoms during pregnancy were among the top queries. Such findings are mirrored by a study which found that 8% of 300 women in Saudi Arabia used ChatGPT for oral health information during pregnancy [29].

Notably, the accuracy of ChatGPT-generated responses regarding cirrhosis was higher in English than in Arabic [30]. The previous study also demonstrated high understandability in both languages, which was in line with the relatively high mean understandability score (89%) in the present study. Highly comprehended information may, in turn, support health literacy, a known facilitator of Arabic health information-seeking on ChatGPT [28]. Furthermore, the average PEMAT score for understandability was comparable to those for ChatGPT-generated English information on reconstructive urology (85%) [29] and cataract surgery (85%) [31]. Similarly, those related to actionability were higher than previous studies (80% compared to 37% and 38%, respectively). Similar findings were observed in responses generated in response to common patient questions about abdominal aortic aneurysms, which notably lacked actionable instructions [2]. With many Arabic patients using LLMs as tools for empowerment [28], those seeking information on dry mouth may find it difficult to act on what they read, as actionability scores were below the recommended levels for 40% of the queries.

It is also necessary to consider increased patient health information-seeking through LLMs, which mirrors their perceived ability to control the disease [32]. Within the oral health context, this can include routine dental care, adopting preventive measures for oral and dental diseases, self-care for oral dryness (e.g., using saliva substitutes and stimulants), and seeking professional help (e.g., for a lump in the mouth or face). Furthermore, the nurse practitioner dental model of a ‘wellness visit’ can be employed for these patients by assessing their medical history related to dryness (e.g., polypharmacy), referring to useful online resources to optimise care plans, monitoring their progress, and scheduling regular follow-up visits with specialists [33]. This may also support value-based oral healthcare delivery by potentially reducing utilisation of advanced oral care services, especially among ageing populations [33].

Several limitations to the present findings remain related to the search engine, reporting bias, a lack of readability assessments, and the linguistic complexities of Modern Standard Arabic (e.g., morpho-phonological principles) and regional dialects for the keywords searched. It is also imperative to address common LLM limitations, such as liability and sources of the information provided, hallucinations, a lack of human-based reasoning and explanations, and language- and culture-related underpinnings [1,5]. Readability assessments were not conducted because accessible, reliable Arabic assessment tools were lacking and differ grammatically from English [18]. This is essential to assess whether the generated text is patient-friendly and sentimentally balanced.

Furthermore, the JAMA quality benchmarks assessment was not applicable for LLM-generated information, as it concerns authorship (who wrote the text, and what are their affiliations?), attributions (where does the information originate from?), disclosure (who owns the information?), and relevance (when was the original information created?). Moreover, the current study’s representations could be carefully addressed by limiting the assessed materials to the first 10. This was evident from the repeated results (*n* = 12) among the top 25 related searches. Also, trends beyond the study’s scope (e.g., diabetes symptoms, dry body, and dry nose) were evident.

The study, however, provided useful insights into the potential role of ChatGPT as a patient education tool for addressing health information needs related to dry mouth. It addressed whether a patient or layperson would find the information comprehensible and whether they could act upon it after reading the generated dry mouth information. The rationale for such work was supported by existing limitations in web-based oral health information and the increased public adoption of ChatGPT as a source of health information [34,35]. Future work may address the performance characteristics of different LLMs towards the reliability/understandability of generated responses allied to oral or dental diseases, adherence to medication regimens, and follow-up healthcare visits. Also, healthcare providers must assess their risk of misinformation and safety, as these sometimes conflict with practice guidelines and available evidence [8]. In oral medicine practice, this may include communicating oral histopathology or imaging reports, providing instructions for the use of oral corticosteroids in oral mucosal disorders, and scheduling visits for potentially malignant oral disorders. It would also be important to address whether this can contribute to better clinician–patient interactions before consultations (e.g., by providing a pre-visit checklist of discussion points) and support value-based healthcare services (e.g., by reducing utilisation of advanced care) [12].

It is not yet evident whether self-care educational sessions may lead to favourable improvements in dryness sensation, saliva quality, and night-time hydration [36]. Thus, future studies should consider clinical studies comparing different educational methods and their long-term impact on clinical outcomes. The clinical outcomes of pre- and post-educational LLM health literacy interventions should also be assessed in relation to oral chronic diseases, such as Sjogren’s syndrome. Given the profound cultural and psychosocial underpinnings of oral and maxillofacial diseases [19], it remains unknown whether LLM-generated health information will consider these factors, particularly in oral diseases.

## 5. Conclusions

With guidance, LLMs could be a useful source for patient education on self-management, symptom recognition, and informed decision-making regarding their own dry mouth care plans. Issues related to which terms to search for, which LLMs to use, linguistic variations in search terms, clinician-assisted review of AI-generated text, and the limitations of these approaches might be addressed with the patient. Developers may consider incorporating visual aids within text for easier adoption and actionability, whereas linguists may consider addressing variations in Arabic language-related prompts compared to English and other languages.

## Figures and Tables

**Table 1:** Patient Education Materials Assessment Tool (PEMAT).

PEMAT: Understandability (*n* = 16) *	PEMAT: Actionability (*n* = 5) *
Evident purpose	Include at least one action
Informative headers	Addresses the reader directly
Concise sections	Actions break down into manageable/clear steps
Using visual cues	Uses a planning checklist or tool
Presents a summary	Presents simple instructions for calculations where available
Presents in a logical sequence	Presents explanation to use charts and graphs
Uses everyday language	Presents visual aids to reinforce understanding of information
Does not expect reader to perform calculations	
Uses illustrations/photos	
Uses non-distracting visual aids	
Uses medical terms to familiarise the reader	
Visual aids whenever feasible	
Visual aids to assist understandability	
Visual aids to reinforce content	
Visual aids with clear headings	
Clear illustrations/photos	
Clear and simple tables	

* Understandability and actionability scores = obtained points/all possible points × 100. Acceptable total scores should be ≥70%.

**Table 2:** The top searched queries related to dry mouth based on Google Trends.

Top Queries *
Causes of Dry Mouth **
Symptoms of Dry Mouth
Treatment of Dry Mouth
Dry Throat
Causes of Dry Mouth ***
Dry Mouth and Throat
Dry Mouth During Sleep
Dry Mouth and Tongue
Dry Lips
Dry Tongue
Dry Mouth During Pregnancy
Symptoms of Diabetes
Dehydration
Dry Mouth Upon Waking Up
Causes of Dry Mouth and Throat
Causes of Dry Throat
Dry Nose

* After removing the redundant terms. ** The searched word without an Arabic glottal stop (hamza). *** The searched word with an Arabic glottal stop.

**Table 3:** Item-wise distribution of PEMAT scores for generated Arabic dry mouth educational content.

PEMAT Items	Searched Terms on ChatGPT									
	Causes of Dry Mouth *	Cause of Dry Mouth	Symptoms of Dry Mouth	Treatment of Dry Mouth	Dry Throat	Causes of Dry Mouth **	Dry Mouth and Throat	What Causes Dry Mouth	Dry Mouth During Sleep	Dry Mouth and Tongue
PEMAT-1 (It makes its purpose completely evident)	1	1	1	1	1	1	1	1	1	1
PEMAT-2 (Presents no distracting information or content)	1	1	1	1	1	1	1	1	1	1
PEMAT-3 (Uses common, everyday language)	1	1	1	1	1	1	1	1	1	1
PEMAT-4 (Medical terms are used only to familiarise the reader with the terms)	1	1	1	1	1	1	1	1	1	1
PEMAT-5 (Uses active voice)	1	1	1	1	1	1	1	1	1	1
PEMAT-6 (Numbers are clear and easy to understand)	N/A	N/A	N/A	N/A	N/A	N/A	N/A	N/A	N/A	N/A
PEMAT-7 (Does not expect the user to perform calculations)	1	1	1	1	1	1	1	1	1	1
PEMAT-8 (Breaks down information into short sections)	1	1	1	1	1	1	1	1	1	1
PEMAT-9 (Sections have informative headers)	1	1	0	0	1	1	1	1	1	1
PEMAT-10 (Presents information in a logical sequence)	1	1	1	1	1	1	1	1	1	1
PEMAT-11 (Provides a summary)	1	1	N/A	N/A	N/A	1	N/A	N/A	N/A	N/A
PEMAT-12 (Uses visual cues to draw attention to key points)	1	1	1	1	1	1	1	1	1	1
PEMAT-15 (Provides visual aids, making content more easily understood)	0	0	0	0	0	0	0	0	0	0
PEMAT-16 (Visual aids reinforce rather than distract from the content)	N/A	N/A	N/A	N/A	N/A	N/A	N/A	N/A	N/A	N/A
PEMAT-17 (Visual aids have clear titles or captions)	N/A	N/A	N/A	N/A	N/A	N/A	N/A	N/A	N/A	N/A
PEMAT-18 (Illustrations and photographs are clear and uncluttered)	N/A	N/A	N/A	N/A	N/A	N/A	N/A	N/A	N/A	N/A
PEMAT-19 (Uses simple tables with short and clear row and column headings)	N/A	N/A	N/A	N/A	N/A	N/A	N/A	N/A	N/A	N/A
Total points	11	11	9	9	10	11	10	10	10	10
Total possible points	12	12	11	11	11	12	11	11	11	11
Understandability score %	91.6	91.6	83.8	83.3	91.6	91.6	90.9	90.9	90.9	90.9
PEMAT-20 (Provides at least one action the user can take)	1	1	1	1	1	1	1	1	1	1
PEMAT-21 (Addresses the user directly when describing actions)	1	1	1	1	1	1	1	1	1	1
PEMAT-22 (Breaks down any action into manageable, explicit steps)	1	1	1	1	1	1	1	1	1	1
PEMAT-23 (Provides a tangible tool whenever it could help the user to act)	1	1	1	1	1	0	0	0	1	0
PEMAT-24 (Provides simple instructions or examples of how to perform calculations)	N/A	N/A	N/A	N/A	N/A	N/A	N/A	N/A	N/A	N/A
PEMAT-25 (Explains how to use the charts, graphs, tables, or diagrams to take actions)	N/A	N/A	N/A	N/A	N/A	N/A	N/A	N/A	N/A	N/A
PEMAT-26 (Uses visual aids whenever possible to make it easier to act on instructions)	0	0	0	0	0	0	0	0	0	0
Total points	4	4	4	4	4	3	3	3	4	3
Total possible points	5	5	5	5	5	5	5	5	5	5
Actionability score %	80	80	80	80	80	60	60	60	80	60

Scoring instructions: Agree = 1, Disagree = 0, Not Applicable = N/A. * The searched term without using an Arabic glottal stop. ** The searched term using an Arabic glottal stop.

## Data Availability

All essential data to support the present study findings are enclosed within the manuscript and Supplementary Files. Those related to analysed Google Trends can be provided upon request from the corresponding author (A.A., aalsoghier@ksu.edu.sa).

## Supplementary Materials

The following supporting information can be downloaded at: https://www.mdpi.com/article/10.3390/healthcare14172681/s1. File S1. The translated content yielded from each search query (*n* = 10); File S2. The generated Arabic content for each searched keyword (*n* = 10); File S3. Google trends searches per country; File S4. Google trends searches overtime.

## Abbreviations

PEMAT	Patient Education Materials Assessment Tool
LLMs	Large language models
ChatGPT	Chat Generative Pretrained Transformer
