# Supplementary material for: Evaluating ChatGPT’s Effectiveness for Arabic Dry Mouth Patient Education
Source: Healthcare (Basel). 2026 Aug 24;14(17):2681. doi: 10.3390/healthcare14172681 (PMC13565665; doi:10.3390/healthcare14172681)
Supplement: Supplementary file 1 [file healthcare-14-02681-s001.zip › Supplementary File S1. The translated content yielded from each search query (n=10).pdf]

Supplementary File S1. The translated content yielded from each search query (n=10).

1. Dry Mouth

Dry mouth (xerostomia) is a condition that occurs when the salivary glands don't produce enough saliva. It can be temporary or persistent and may cause difficulty eating, speaking, or swallowing, and can affect oral health.

Causes of Dry Mouth

1 .General Dehydration

- Not drinking enough water.
- Fluid loss due to excessive sweating.
- Diarrhea or vomiting.
- Fever and high temperature.

2 .Medications

- The most common causes include:
- Antihistamines (allergy medications).
- Antidepressants.
- Some painkillers and sedatives.
- Blood pressure medications.
- Diuretics.
- Some medications for neurological conditions.

3 .Mouth Breathing

- Dry mouth is exacerbated by:
- Nasal congestion.
- Nasal allergies.
- Deviated septum.
- Snoring during sleep.

4 .Diseases and Health Conditions

- Diabetes.
- Sjögren's syndrome.
- Thyroid disorders.
- Kidney disease.
- Some autoimmune and neurological diseases.

5 .Stress and Anxiety
Stress and anxiety can reduce saliva production and cause a dry mouth.

6. Smoking and Alcohol
These reduce saliva flow and increase the feeling of dryness and burning.

7. Radiation Therapy

Especially to the head and neck area, as it can affect the salivary glands and reduce saliva production.

8. Aging

Some older adults experience decreased saliva production, especially with the use of multiple medications.

Simple Treatments for Dry Mouth

- Drink water regularly.
- Avoid coffee, tea, and carbonated drinks as they worsen dehydration.
- Chew sugar-free gum to stimulate saliva production.
- Use saliva substitutes (available at pharmacies).
- Humidify the air using a humidifier.
- Treat the underlying cause (such as adjusting medication or treating nasal congestion).

If you wish, please tell me your age, symptoms, and any medications you are taking so I can determine your likely cause and suggest appropriate treatment.

2. Causes of Dry Mouth

There are several common causes of dry mouth, some of which may be temporary, while others may indicate an underlying health condition requiring treatment. The most important causes include:

1 .Reduced Saliva Production (True Dry Mouth)

- General dehydration due to insufficient water intake.
- Breathing through the mouth instead of the nose, especially during sleep.
- Dry air and the use of air conditioning.

2 .Medications

Medications are among the most common causes and include:

- Allergy medications (anthistamines).
- Antidepressants and anti-anxiety medications.
- Some painkillers.
- Blood pressure medications (such as diuretics).
- Some medications for chronic medical conditions.

3. Medical Conditions

- Diabetes (especially when blood sugar is high, as this causes thirst and dry mouth).
- Thyroid disorders.
- Sjögren's syndrome, which causes dry mouth and eyes.
- Some neurological diseases that affect the salivary glands.

4 .Lifestyle Factors

- Smoking.
- Alcohol.
- Excessive caffeine intake (coffee, tea, energy drinks).

5 .Salivary gland problems

- Blockage or inflammation of the salivary glands.
- Radiation therapy of the head and neck area.

When should you see a doctor?

- It is advisable to see a doctor if your dry mouth:
- Consists of a prolonged period.
- Causes difficulty swallowing or speaking.
- Is accompanied by dry eyes or skin.
- Is it related to new medications.
- Causes bad breath or frequent tooth decay.

If you wish, please tell me: Is the dryness persistent? What medications are you taking? Do you have any chronic conditions such as diabetes?

3. Symptoms of Dry Mouth

Dry mouth (xerostomia) may develop gradually or appear suddenly. Common symptoms include:

- Persistent thirst.
- Difficulty swallowing or the sensation that food is stuck in the throat.
- Cracked lips and dryness around the mouth.
- Bad breath.
- A tongue that may stick to the roof of the mouth.
- Difficulty speaking or a rough voice.
- Changes in taste perception or partial loss of taste.
- Increased risk of tooth decay or recurrent gum inflammation due to low salivation.
- Burning sensation of the tongue.

Common Causes of Dry Mouth

- Certain medications (antidepressants, antihistamines, and blood pressure medication…).
- Stress and anxiety.
- Mouth breathing.
- Dehydration due to inadequate fluid intake.
- Diabetes mellitus.
- Radiation therapy to the head and neck region.
- Disorders affecting the salivary glands.

When to Seek Medical Advice?

- Persists for an extended period.
- Affects eating, swallowing, or speaking.
- Is accompanied by pain, swelling of the salivary glands, or recurrent dental decay.

If you would like, I can help you identify possible causes of dry mouth and suggest appropriate treatment options based on your condition.

4. Dry Mouth Treatment

Dry mouth (xerostomia) is a common condition that occurs when the salivary glands do not produce enough saliva. It can be uncomfortable and may affect quality of life. The following measures can help relieve dry mouth symptoms:

1 .Drink water regularly
It is important to drink adequate amounts of water throughout the day. Proper hydration helps stimulate saliva production and reduces the feeling of dryness in the mouth.

2 .Use oral moisturizers
There are products specifically designed for dry mouth, such as moisturizing sprays, gels, and oral rinses (for example, xerostom). These products help keep the mouth moist and improve comfort.

3 .Chew sugar-free gum or use sugar-free candy
Chewing sugar-free gum or consuming sugar-free candies can stimulate saliva production and help alleviate dryness.

4 .Use saline solutions or specialized mouth rinses
Saline rinses and mouthwashes formulated for dry mouth may help maintain oral moisture and improve symptoms.

5 .Use a humidifier
Using a humidifier, especially in dry environments or during sleep, may help increase moisture in the air and reduce dry mouth symptoms.

6 .Avoid salty or spicy foods
Salty and spicy foods can worsen dry mouth symptoms, so reducing their intake may be beneficial.

7 .Stop smoking
Smoking can contribute to or worsen dry mouth. Quitting smoking is recommended for people experiencing persistent oral dryness.

8 .Consult a doctor
If dry mouth persists, it may indicate an underlying medical condition such as diabetes or hormonal changes. A healthcare professional can help determine the cause and recommend appropriate treatment.

Do you suffer from persistent dry mouth, or is it only a temporary symptom?

5. Dry Throat

Dry throat can occur for a variety of reasons, including:

1. Mouth breathing: especially during sleep or when the nose is blocked.
2. General dehydration: lack of fluids or not drinking enough water.
3. Dry or cold weather: dry or cold air may cause dryness of the throat and nose.
4. Exposure to polluted air: such as dry air in air-conditioned environments or cigarette smoke.
5. Viral or bacterial infections: such as the common cold or throat infections.
6. Use of certain medications: such as allergy medications or decongestants, which may cause throat dryness as a side effect.
7. Allergies: allergic reactions may contribute to throat dryness.

Ways to relieve dry throat

- Drink water regularly.
- Use a humidifier if the air is dry.
- Gargle with warm salt water.
- Avoid smoking and exposure to polluted air.

If dry throat persists for an extended period or is accompanied by symptoms such as severe pain or difficulty swallowing, medical advice should be sought.

6. Causes of Dry Mouth

Dry mouth is a condition that occurs when the salivary glands do not produce enough saliva. It can be uncomfortable and may make speaking, eating, or swallowing more difficult. Common causes include:
1. Medications
Several medications are known to cause dry mouth, including:

- Antidepressants.
- Antihistamines.
- Allergy medications.
- Medications used to treat high blood pressure.

2. Dehydration
Not drinking enough fluids, or losing fluids through excessive sweating or diarrhea, can result in dry mouth.

3. Salivary gland problems
Conditions affecting the salivary glands, such as infections or tumors, may reduce saliva production and lead to dry mouth.

4. Smoking
Smoking can significantly contribute to dry mouth and negatively affect overall oral health.

5. Aging
As people get older, salivary gland function may decline, and age-related diseases (such as diabetes) may affect saliva production.

6 .Stress and anxiety
Psychological stress and chronic anxiety may contribute to dry mouth through their effects on the nervous system.

7 .Certain health conditions such as diabetes, high blood pressure, HIV/AIDS or Parkinson's disease may also lead to dry mouth.

8 .Mouth Breathing
Breathing through the mouth, especially during sleep due to nasal blockage, can cause dry mouth.

9 .Radiation Therapy
People receiving radiation therapy to the head or neck may develop dry mouth because the treatment can affect the salivary glands.

10 .Alcohol and Caffeine Consumption
Alcohol and caffeine may contribute to dry mouth by reducing hydration and decreasing saliva production.

If dry mouth is persistent or accompanied by other problems such as tooth decay or oral infections, medical or dental evaluation is recommended to identify the underlying cause and determine the appropriate treatment.

7. Dry Mouth and Dry Throat

Dry mouth and dry throat can be very uncomfortable, and there are several possible causes that may contribute to this problem. The most common causes include:
1 .Insufficient fluid intake: not drinking enough water can lead to dryness of both the mouth and throat.

2 .Mouth breathing: if you breathe through your mouth rather than your nose, especially while sleeping, it may cause dryness in the mouth and throat.

3 .Medications: certain medications, such as antihistamines and blood pressure medications, can cause dry mouth.

4 .Exposure to dry air: this is particularly common in environments with air conditioning or indoor heating.

5 .Medical conditions: some conditions, including diabetes and upper respiratory tract infections, may contribute to dry mouth and throat.

6 .Anxiety or stress: anxiety and stress may reduce saliva production, which can result in dry mouth.

If the problem is persistent or affects daily activities, it is advisable to consult a healthcare professional to identify the underlying cause and receive appropriate treatment. Symptoms can often be relieved by drinking more water and using a humidifier.

Do you suffer from this problem on an ongoing basis, or has it appeared recently?

8. What Causes Dry Mouth?

Dry mouth occurs when the salivary glands do not produce enough saliva. Saliva is important for keeping the mouth moist, helping digestion, and protecting the teeth from decay and the gums from infection. There are several possible causes of dry mouth:

1 .Dehydration and inadequate fluid intake:
Not drinking enough water or other fluids can lead to dry mouth, especially during hot weather or while exercising.

2 .Medications:
Some medications can cause dry mouth as a side effect, including antidepressants, antihistamines, blood pressure medications, and certain allergy medications.

3 .Diseases:
Some health conditions may contribute to dry mouth, such as diabetes, rheumatoid arthritis, salivary gland disorders, and Sjögren's syndrome.

4 .Nerve injuries or damage:
Injuries or conditions that affect the nerves responsible for saliva production may result in dry mouth.

5 .Smoking or tobacco use:
Smoking or chewing tobacco can reduce saliva production and increase the likelihood of dry mouth.

6 .Anxiety and stress:
Emotional stress and anxiety can contribute to dry mouth symptoms.

7 .Hormonal changes:
Hormonal fluctuations, such as those occurring during pregnancy or with aging, may affect saliva production.

8 .Mouth breathing:
Breathing through the mouth, often due to nasal congestion or habitual mouth breathing, can cause dryness of the mouth.

If dry mouth persists or occurs together with other symptoms, it is advisable to consult a healthcare professional to identify the underlying cause and receive appropriate treatment.

9. Dry Mouth During Sleep

Dry mouth during sleep is a common problem and can be quite uncomfortable. It usually occurs when saliva flow decreases during the night, leading to a feeling of dryness in the mouth upon waking. There are several possible causes of this condition.

1 .Mouth breathing: if you breathe through your mouth instead of your nose while sleeping, this can cause dry mouth. This may happen because of nasal congestion, allergies, or other breathing-related issues.

2 .Certain medications: some medications can cause dry mouth as a side effect such as antihistamines, antidepressants, blood pressure medications and certain allergy medications.

3 .General dehydration: Not drinking enough water throughout the day may lead to dry mouth at night.

4 .Smoking and alcohol: Smoking or drinking alcohol before bedtime can reduce saliva production and increase dry mouth.

5 .Medical conditions: Certain health conditions such as diabetes and salivary gland disorders may cause dry mouth

Tips to relieve dry mouth during sleep

- Breathe through your nose: If you suffer from nasal blockage, try to address the underlying cause. Nasal sprays or medical consultation may help improve nasal breathing.
- Drink more water during the day: Make sure you consume enough water throughout the day, especially before bedtime.
- Use a humidifier: If the air in your room is dry, using a humidifier may help reduce dry mouth symptoms.
- Avoid smoking and alcohol before bed: Reducing or avoiding tobacco and alcohol use before sleeping may lessen dryness overnight.
- Use dry-mouth products: Specialized mouth rinses, sprays, and moisturizing products designed for dry mouth may help keep the mouth comfortable during the night.

If dry mouth continues for a long time, becomes severe, or occurs together with other symptoms, it is advisable to consult a physician or dentist to determine the cause and receive appropriate treatment.

10. Dry Mouth and Tongue

Dry mouth and tongue can be caused by several factors and may be accompanied by other symptoms such as difficulty speaking, swallowing, or a persistent feeling of thirst. One of the most common causes is a lack of fluids in the body, which is why maintaining adequate hydration is important.

1 .Not drinking enough water: Insufficient water and fluid intake can lead to dryness of the mouth and tongue, particularly during hot weather or periods of increased fluid loss.

2 .Mouth breathing: Breathing through the mouth instead of the nose, especially during sleep, can contribute to dryness of the mouth and tongue.

3 .Certain medications: Some medications such as antihistamines, blood pressure medications, and antidepressants may cause dry mouth as a side effect.

4 .Salivary gland problems: Such as inflammation of the salivary glands or disorders like “Sjögren’s syndrome” may affect the glands that produce saliva.

5 .Stress and anxiety: Psychological stress may affect saliva flow and causing dry mouth.

6 .Smoking: Smoking can contribute to dry mouth by reducing saliva production.

7 .Other health conditions: Such as diabetes and thyroid gland disorders.

If dry mouth persists for a long period or is accompanied by other symptoms, it is recommended to consult a doctor to determine the exact cause. General recommendations include drinking water regularly and using oral moisturizers or mouth rinses specifically designed for dry mouth.
