# Supplementary material for: Evaluating ChatGPT’s Effectiveness for Arabic Dry Mouth Patient Education
Source: Healthcare (Basel). 2026 Aug 24;14(17):2681. doi: 10.3390/healthcare14172681 (PMC13565665; doi:10.3390/healthcare14172681)
Supplement: Supplementary file 1 [file healthcare-14-02681-s001.zip › Supplementary File S2. The generated Arabic content for each searched keyword (n=10).pdf]

اسباب جفاف الفم

إليك **أهم أسباب جفاف الفم (جفاف الحلق أو نقص اللعاب)** بشكل واضح ومبسط:

1) الجفاف ونقص السوائل

- عدم شرب كمية كافية من الماء.
- فقدان السوائل بسبب الحرّ الشديد، التعرّق، الإسهال أو القيء.

2) الأدوية

تُعدّ الأدوية من أكثر الأسباب شيوعًا، مثل:

- مضادات الاكتئاب والقلق.
- أدوية الحساسية (مضادات الهيستامين).
- أدوية ارتفاع الضغط (مدرات البول).
- أدوية الاحتقان.
- بعض المسكنات والمهدئات.

3) التنفس من الفم

- بسبب انسداد الأنف (حساسية – انحراف حاجز الأنف – لحمية).
- خلال النوم، خصوصًا عند الشخير.

4) بعض الأمراض

- السكري (ارتفاع السكر يسبب العطش وجفاف الفم).
- الغدة الدرقية (خمولها أو فرط نشاطها).
- متلازمة شوغرن (يهاجم الجهاز المناعي الغدد اللعابية).
- أمراض الكلى.

5) التوتر والقلق

القلق يثبّط إفراز اللعاب ويسبب إحساسًا بجفاف الفم.

6) التدخين

التدخين والشيشة يقللان تدفق اللعاب ويهيّجان الفم والحنجرة.

7) العلاج الإشعاعي أو الكيماوي

خاصةً علاج سرطانات الرأس والرقبة.

8) التقدم في العمر

يقل إفراز اللعاب طبيعيًا مع التقدم في السن، إضافةً إلى كثرة الأدوية.

علاج جفاف الفم بشكل مبسط

- شرب الماء بانتظام.
- تجنب القهوة والشاي والمشروبات الغازية لأنها تزيد الجفاف.
- مضغ علكة خالية من السكر لتحفيز اللعاب.
- استخدام بدائل اللعاب (تُباع في الصيدليات).
- ترطيب الجو باستخدام جهاز ترطيب.
- علاج السبب الأساسي (مثل تعديل دواء أو علاج انسداد الأنف).

إذا رغبت، أخبرني **عمرك، الأعراض، والأدوية التي تستخدمها** لأحدد السبب المرجّح لديك وأقترح علاجًا مناسبًا.

سبب جفاف الفم

هناك عدة **أسباب شائعة لجفاف الفم**، وقد يكون بعضها بسيطًا ومؤقتًا بينما يشير بعضها الآخر إلى حالة صحية تحتاج لعلاج. من أهم الأسباب:

1) نقص إفراز اللعاب (جفاف فم حقيقي)

- الجفاف العام في الجسم (قلة شرب الماء).
- التنفس عبر الفم بدل الأنف، خاصة أثناء النوم.
- الجو الجاف واستخدام المكيفات.

2) الأدوية

من أكثر الأسباب شيوعًا، وتشمل:

- أدوية الحساسية (مضادات الهيستامين).
- مضادات الاكتئاب والقلق.
- مسكنات معينة.
- أدوية ارتفاع الضغط (مثل المدرّات).
- أدوية نزلات البرد.

3) حالات طبية

- **السكري** (ارتفاع السكر يسبب عطشًا وجفافًا).
- **اضطرابات الغدة الدرقية.**
- **متلازمة شوغرِن** (تسبب جفاف الفم والعينين).
- أمراض عصبية تؤثر على الغدد اللعابية.

4) عوامل نمط الحياة

- التدخين.
- الكافيين الزائد (قهوة، شاي، مشروبات طاقة).
- الكحول.

5) مشاكل الغدد اللعابية

- انسداد أو التهاب الغدد اللعابية.
- العلاج الإشعاعي لمنطقة الرأس والرقبة.

متى يجب زيارة الطبيب؟

راجع طبيبًا إذا كان الجفاف:

- مستمرًا لفترة طويلة.
- يسبب صعوبة في البلع أو الكلام.
- يصاحبه جفاف العين أو الجلد.
- مرتبطًا بأدوية جديدة.
- يسبب رائحة فم كريهة أو تسوُّس متكرر.

إذا رغبت، يمكنني مساعدتك أكثر

✔️ أخبرني: هل الجفاف مستمر؟ وهل تستخدم أدوية معينة أو تعاني من أمراض مزمنة مثل السكري؟

اعراض جفاف الفم

أعراض جفاف الفم (ドry mouth / Xerostomia) قد تظهر بشكل تدريجي أو مفاجئ، وتشمل عادةً:

الأعراض الشائعة

- **الإحساس بالعطش المستمر**
- **صعوبة بلع الطعام** أو الشعور بأن الطعام عالق بالحلق
- **تشققات الشفاه** وجفاف الجلد حول الفم
- **رائحة فم كريهة**
- **التصاق اللسان بسقف الحلق**
- **جفاف الحلق** وخاصة أثناء الليل
- **صعوبة في التحدث** أو الصوت المتخشب
- **تغير في التذوق** أو فقدان جزئي لحاسة التذوق
- **زيادة تسوس الأسنان** أو التهابات اللثة نتيجة نقص اللعاب
- **إحساس بالحرقان في اللسان**

أسباب شائعة لجفاف الفم

- الأدوية (مضادات الاكتئاب، مضادات الحساسية، أدوية ضغط الدم...)
- التوتر والقلق
- التنفس عبر الفم
- الجفاف الناتج عن قلة السوائل
- مرض السكري
- العلاج الإشعاعي للرأس والرقبة
- مشاكل الغدد اللعابية

متى يجب زيارة الطبيب؟

- إذا كان جفاف الفم مستمرًا
- إذا كان يؤثر على الأكل أو الكلام
- إذا ترافق مع ألم، تورم الغدد، أو تسوس متكرر

إذا أحببت، يمكنني مساعدتك في معرفة **طرق علاج جفاف الفم** أو **الأسباب المحتملة حسب حالتك**.

علاج جفاف الفم

جفاف الفم (أو *xerostomia* ت) هو حالة شائعة تحدث عندما لا تنتج الغدد اللعابية كمية كافية من اللعاب. يمكن أن يكون مزعجًا ويؤثر على جودة الحياة. إليك بعض العلاجات التي يمكن أن تساعد في تخفيف جفاف الفم:

1. **شرب الماء بانتظام:**
- من المهم شرب كميات كافية من الماء طوال اليوم لتحفيز إنتاج اللعاب وتخفيف جفاف الفم.
2. **استخدام مرطبات الفم:**
- توجد منتجات مثل جل أو رذاذ مرطب للفم (مثل "Xerostom") التي تساعد في ترطيب الفم وتخفيف الإحساس بالجفاف.
3. **مضغ العلكة أو الحلوى الخالية من السكر:**
- مضغ العلكة الخالية من السكر أو تناول حلوى خالية من السكر يمكن أن يساعد في تحفيز إنتاج اللعاب. بعض الأنواع تحتوي على مواد مثل *الزيتون* أو *السوربيتول* التي تحفز الغدد اللعابية.
4. **استخدام محلولات ملحية:**
- يمكن أن تساعد المحاليل الملحية أو غسول الفم المخصص للحفاظ على رطوبة الفم في تحسين الحالة.
5. **استخدام مرطبات هواء:**
- في الأجواء الجافة، يمكن أن يكون من المفيد استخدام جهاز ترطيب الهواء في الغرفة.
6. **تجنب الأطعمة المالحة أو الحارة:**
- الأطعمة الحارة أو المالحة يمكن أن تزيد من جفاف الفم، لذا يفضل تجنبها.
7. **الإقلاع عن التدخين:**
- التدخين يمكن أن يزيد من جفاف الفم، لذا ينصح بالإقلاع عنه إذا كنت مدخنًا.
8. **مراجعة الطبيب:**
- إذا كان جفاف الفم مستمرًا، فقد يكون علامة على مشكلة صحية أخرى مثل مرض السكري أو التغيرات الهرمونية. لذلك، من الأفضل مراجعة الطبيب أو طبيب الأسنان لتحديد السبب الكامن ومعالجة المشكلة.

هل تعاني من جفاف الفم بشكل مستمر، أم أنه عرض مؤقت؟

جفاف الحلق

جفاف الحلق يمكن أن يحدث بسبب عدة أسباب، ومنها:

1. **التنفس عن طريق الفم:** خاصة أثناء النوم أو عندما تكون الأنف مسدودة.
2. **الجفاف العام في الجسم:** نقص السوائل أو عدم شرب كميات كافية من الماء.
3. **الطقس الجاف أو البارد:** يمكن أن يؤدي إلى جفاف الحلق والأنف.
4. **التعرض للهواء الملوث:** مثل الهواء الجاف في الأماكن المغلقة أو دخان السجائر.
5. **العدوى الفيروسية أو البكتيرية:** مثل الزكام أو التهاب الحلق.
6. **استخدام الأدوية:** بعض الأدوية، مثل أدوية الحساسية أو مضادات الاحتقان، قد تسبب جفاف الحلق كأثر جانبي.
7. **الحساسية:** تفاعلات مع المواد المسببة للحساسية قد تسبب جفاف الحلق.

لتخفيف جفاف الحلق، يمكنك:

- شرب الماء بانتظام.
- استخدام مرطب الهواء إذا كان الجو جافًا.
- الغرغرة بماء دافئ وملح.
- تجنب التدخين أو التعرض للهواء الملوث.

إذا استمر الجفاف لفترة طويلة أو كان مصحوبًا بأعراض أخرى، مثل ألم شديد أو صعوبة في البلع، فقد يكون من الأفضل استشارة الطبيب.

أسباب جفاف الفم

جفاف الفم (أو ما يُسمى *بالزلاق* في بعض الأحيان) هو حالة تحدث عندما لا تنتج الغدد اللعابية كميات كافية من اللعاب. وهذا يمكن أن يكون مزعجًا للغاية وقد يؤدي إلى صعوبة في الكلام، الأكل، أو حتى ابتلاع الطعام. هناك عدة أسباب قد تؤدي إلى جفاف الفم، ومنها:

1. الأدوية

بعض الأدوية تعتبر من الأسباب الشائعة لجفاف الفم، مثل:

- أدوية مضادة للاكتئاب
- أدوية مضادة للهستامين
- أدوية لعلاج ضغط الدم
- أدوية لعلاج الحساسية

2. الجفاف (Dehydration)

قلة شرب السوائل أو فقدان السوائل بسبب التعرق المفرط، الإسهال، أو التقيؤ قد يسبب الجفاف، مما يؤدي إلى جفاف الفم.

3. مشاكل الغدد اللعابية

أي مشكلة تؤثر في الغدد اللعابية مثل التهابات أو أورام قد تؤدي إلى نقص في إفراز اللعاب. بعض الأمراض مثل **متلازمة شوغرن** (مرض مناعي) قد تؤثر بشكل كبير على إنتاج اللعاب.

4. التدخين

التدخين أو استخدام التبغ يمكن أن يؤدي إلى جفاف الفم بشكل مباشر ويؤثر على صحة الفم بشكل عام.

5. التقدم في السن

مع التقدم في العمر، قد تقل قدرة الغدد اللعابية على إنتاج اللعاب. كما أن بعض الأمراض التي تصيب كبار السن (مثل مرض السكري) قد تؤثر أيضًا على إنتاج اللعاب.

6. التوتر والقلق

الضغط النفسي أو التوتر المزمن يمكن أن يسبب جفاف الفم نتيجة لتأثيره على النظام العصبي.

7. مشاكل صحية معينة

بعض الحالات الصحية مثل **مرض السكري**، **ارتفاع ضغط الدم**، **الإيدز**، أو **مرض باركنسون** قد تؤدي أيضًا إلى جفاف الفم.

8. التنفس عن طريق الفم

التنفس عبر الفم أثناء النوم (بسبب انسداد الأنف مثلاً) قد يسبب جفاف الفم.

9. العلاج الإشعاعي

الأشخاص الذين يخضعون للعلاج الإشعاعي في منطقة الرأس أو الرقبة قد يعانون من جفاف الفم بسبب تأثيره على الغدد اللعابية.

10. تناول الكحول أو الكافيين

الكحول والكافيين يمكن أن يسببان جفاف الفم لأنه يؤثر على ترطيب الجسم ويقلل من إنتاج اللعاب.

إذا كنت تعاني من جفاف الفم بشكل مستمر أو مصحوب بمشاكل أخرى مثل تسوس الأسنان أو التهابات في الفم، من المهم استشارة الطبيب أو طبيب الأسنان لتحديد السبب والعلاج المناسب.

جفاف الفم والحلق

جفاف الفم والحلق قد يكون مزعجًا جدًا، وهناك العديد من الأسباب المحتملة التي قد تؤدي إلى هذه المشكلة. من أبرز الأسباب:

1. **قلة تناول السوائل**: عدم شرب كمية كافية من الماء قد يؤدي إلى جفاف الفم والحلق.
2. **التنفس عبر الفم**: إذا كنت تتنفس من فمك بدلاً من أنفك، خاصة أثناء النوم، فقد يسبب ذلك جفافًا في الفم والحلق.
3. **الأدوية**: بعض الأدوية مثل مضادات الهيستامين أو أدوية ضغط الدم يمكن أن تسبب جفاف الفم.
4. **التعرض للهواء الجاف**: خاصة في الأماكن ذات التكييف أو التدفئة المكثفة.
5. **الأمراض**: بعض الأمراض مثل السكري أو التهابات الجهاز التنفسي العلوي يمكن أن تؤدي إلى جفاف الفم والحلق.
6. **التوتر أو القلق**: يمكن أن يؤدي القلق أو التوتر إلى تقليل إفراز اللعاب، مما يسبب جفافًا في الفم.

إذا كانت هذه المشكلة مستمرة أو تؤثر على حياتك اليومية، فمن الأفضل زيارة الطبيب لتحديد السبب وتقديم العلاج المناسب. في الحالات البسيطة، يمكنك تجنب جفاف الفم والحلق عن طريق شرب المزيد من الماء، واستخدام مرطبات الهواء أو بخاخات الفم.

هل تعاني من هذه المشكلة بشكل مستمر، أو هل ظهرت مؤخرًا؟

ما سبب جفاف الفم

جفاف الفم (أو "القلس الجاف") يحدث عندما لا تنتج الغدد اللعابية في الفم ما يكفي من اللعاب. اللعاب مهم لترطيب الفم، ويساعد على الهضم ويحمي الأسنان من التسوس واللثة من الالتهابات. هناك عدة أسباب قد تؤدي إلى جفاف الفم، من أبرزها:

1. **الراحة في الفم أو قلة تناول السوائل:**
عدم شرب كميات كافية من الماء قد يؤدي إلى جفاف الفم، خصوصًا في أوقات الطقس الحار أو عند ممارسة التمارين الرياضية.
2. **الأدوية:**
بعض الأدوية، مثل أدوية مضادة للاكتئاب، مضادات الهيستامين، أدوية ضغط الدم، وبعض أدوية الحساسية، قد تسبب جفاف الفم كأثر جانبي.
3. **الأمراض:**
بعض الحالات الصحية مثل السكري، أو التهاب المفاصل الروماتويدي، أو اضطرابات الغدد اللعابية مثل متلازمة شوغرن قد تؤدي إلى جفاف الفم.
4. **الإصابة أو الأضرار العصبية:**
إصابات الوجه أو الرقبة التي تؤثر على الأعصاب المسؤولة عن إفراز اللعاب قد تسبب جفاف الفم.
5. **التدخين أو استخدام التبغ:**
التدخين أو مضغ التبغ يمكن أن يقلل من إفراز اللعاب ويسبب جفاف الفم.
6. **القلق والتوتر:**
التوتر النفسي قد يؤدي إلى جفاف الفم، حيث يمكن أن يؤثر على الغدد اللعابية.
7. **التغيرات الهرمونية:**
التغيرات الهرمونية التي تحدث خلال فترة الحمل أو سن اليأس يمكن أن تؤثر أيضًا على إنتاج اللعاب.
8. **التنفس عبر الفم:**
التنفس عن طريق الفم، سواء بسبب انسداد الأنف أو العادات الشخصية، يمكن أن يسبب جفاف الفم.

إذا كنت تشعر بجفاف في الفم بشكل مستمر أو مع أعراض أخرى، يفضل استشارة طبيب لتحديد السبب ومعالجة المشكلة.

جفاف الفم اثناء النوم

جفاف الفم أثناء النوم هو مشكلة شائعة ويمكن أن تكون مزعجة جدًا. يحدث عادة عندما يقل تدفق اللعاب في الفم أثناء النوم، مما يسبب شعورًا بالجفاف في الفم. هناك عدة أسباب محتملة لهذه المشكلة:

1. **التنفس عبر الفم**: إذا كنت تتنفس من خلال فمك أثناء النوم بدلاً من الأنف، فإن ذلك قد يؤدي إلى جفاف الفم. قد يحدث هذا بسبب انسداد الأنف أو مشاكل تنفسية مثل الشخير أو الحساسية.
2. **استخدام بعض الأدوية**: بعض الأدوية مثل مضادات الهيستامين، أدوية الاكتئاب، أدوية ضغط الدم، وأدوية الحساسية يمكن أن تسبب جفاف الفم كأثر جانبي.
3. **الجفاف العام**: عدم شرب كمية كافية من الماء خلال اليوم قد يؤدي إلى جفاف الفم أثناء النوم.
4. **التدخين والكحول**: التدخين أو شرب الكحول قبل النوم يمكن أن يقلل من إفراز اللعاب ويزيد من جفاف الفم.
5. **مشاكل صحية**: بعض الحالات الصحية مثل مرض السكري أو اضطرابات الغدد اللعابية قد تسبب جفاف الفم.

نصائح للتخفيف من جفاف الفم أثناء النوم:

- **تنفس من الأنف**: حاول التنفس عبر الأنف أثناء النوم. إذا كنت تعاني من انسداد في الأنف، قد يكون من المفيد استخدام بخاخات الأنف أو استشارة الطبيب.
- **شرب الماء قبل النوم**: تأكد من أنك تشرب كمية كافية من الماء خلال اليوم، خاصة قبل النوم.
- **استخدام مرطب الجو**: إذا كنت في منطقة جافة، قد يساعد استخدام جهاز ترطيب للهواء على تقليل جفاف الفم.
- **تجنب التدخين والكحول**: من الأفضل تجنب التدخين وشرب الكحول في الساعات التي تسبق النوم.
- **استخدام غسول فم أو جل لترطيب الفم**: هناك بعض الغسولات أو الجل المخصص للفم التي تساعد في ترطيبه أثناء الليل.

إذا استمر الجفاف بشكل مزمن أو كان مصحوبًا بأعراض أخرى، قد يكون من الأفضل زيارة الطبيب أو طبيب الأسنان لتحديد السبب وعلاجه بشكل دقيق.

جفاف الفم واللسان

جفاف الفم واللسان يمكن أن يكون نتيجة لعدة أسباب، وقد يترافق مع أعراض أخرى مثل صعوبة في التحدث أو البلع، أو شعور بالعطش المستمر. الأسباب المحتملة تشمل:

1. **قلة شرب الماء**: من أبرز الأسباب لجفاف الفم واللسان هو نقص السوائل في الجسم، لذا يفضل زيادة تناول الماء والسوائل.
2. **التنفس عبر الفم**: التنفس من الفم بدلاً من الأنف، خصوصًا أثناء النوم، يمكن أن يؤدي إلى جفاف الفم.
3. **أدوية معينة**: بعض الأدوية، مثل مضادات الهيستامين، أدوية ضغط الدم، أو أدوية مضادة للاكتئاب، يمكن أن تؤدي إلى جفاف الفم كأثر جانبي.
4. **مشاكل في الغدد اللعابية**: مثل التهاب الغدد اللعابية أو حالات مثل "متلازمة سجوجرن"، التي تؤثر على الغدد المسؤولة عن إنتاج اللعاب.
5. **التوتر والقلق**: قد يؤثر التوتر النفسي على تدفق اللعاب ويؤدي إلى جفاف الفم.
6. **التدخين**: يمكن أن يتسبب التدخين في تقليل إفراز اللعاب، مما يؤدي إلى جفاف الفم.
7. **مشاكل صحية أخرى**: مثل السكري أو مشاكل في الغدة الدرقية.

إذا استمر جفاف الفم لفترة طويلة أو كان مصحوبًا بأعراض أخرى، يُفضل استشارة الطبيب للتأكد من السبب الدقيق. من النصائح العامة يمكن محاولة شرب الماء بانتظام، واستخدام مرطبات الفم أو غسولات الفم المخصصة لذلك.
